# Supplementary material for: Agility and Sustainability: A Qualitative Evaluation of COVID-19 Non-pharmaceutical Interventions in the UK Logistics Sector
Source: Front Public Health. 2022 Jun 1;10:864506. doi: 10.3389/fpubh.2022.864506 (PMC9198245; doi:10.3389/fpubh.2022.864506)
Supplement: Supplementary file 2 [file Data_Sheet_1.pdf]

Manuscript: Agility and sustainability: A qualitative evaluation of COVID-19 Non-pharmaceutical Interventions (NPIs) in the UK logistics sector

Additional file 2:

Interview schedule round 1

Q1.1 Under normal circumstances (i.e. pre-March), what is the typical driver shift length and number of shifts per week? Are onsite staff shifts similar?

Q2.2 How has the COVID-19 pandemic affected driver hours? Has this been substantially different to the effects on onsite staff?

Q3 Can we first check out some of the details of your delivery protocol? (Sub-questions customised to companies according to the type of goods delivered)

Q3.1 Under normal circumstances, roughly what fraction of deliveries large items needing two persons?

Q3.2 Before March, how were drivers paired for two-person tasks? Would they be paired for the whole day? Are there any measures being put in place to keep them social distancing?

Q3.3 What type of vehicles are normally used?

Q3.4 Do you use external couriers for any deliveries? Has this changed during the pandemic?

Q3.5 What shared spaces are there at the depots or warehouses (e.g. shared offices, bathrooms, canteens, break rooms)? Under normal circumstances, would onsite staff regularly interact with delivery drivers? Would there usually be team-meetings/handover sessions at the beginning or end of shifts?

Q3.6 What social distancing measures have been put in warehouses/depots, including shift pattern changes. Could you give a little more detail about the shift changes?

Q3.7 Are you providing or recommending the use of facemasks or other PPE to the drivers? Has there been any government guidance on this point? How do you check whether the instructions are properly followed?

Q3.8 The NHS is providing free tests for frontline workers. Do you encourage your delivery workers to take such tests? Is there a policy about taking test? Do you ask them to inform you the results?

Q4 How do you keep your frontline workers updated of the latest information about COVID-19? And effectively communicate the change of practices to your workers and customers? Such as methods, frequencies. How do you check that the messages are received and understood?

Q5 You probably have covered some aspects of this question while we talked about the delivery protocol and communications just now, just in case we have missed anything. So how has the COVID-19 outbreak affected your company in general? Such as volume of work, number of employees, workloads, working hours, type of products / deliveries and mode of delivery

Q6 When we plan for potential future outbreaks, what scenarios (or conditions or risk factors) you consider the most important and must be taken into account?

Q7 We are gathering information to assess companies' risk management strategies in this sector during the pandemic. Do you have a summary timeline of your responses to the pandemic that you could share with us?

Q8 What other activities have your company carried out in response to COVID-19 and are relevant to transmission or protection of workers? Is there any other information that might be useful for our research that we have not asked about?

Interview schedule round 2

Q1 This pandemic has lasted longer than expected and we have been through a lot of changes over the past 6 months. Since then, have you put into place new risk mitigation measures? Have you changed any of the measures in light of the new conditions? Have you seen surge of cases among workforce because of the new variants? (if yes, how did you respond to that? What measures do you think have been the most useful to contain it?)

Q2 The peak period has lasted so long, how are you and colleagues coping with a very high workload for such a long time? Has the workload changed since the relaxation of lockdown measures and reopening of shops/pubs?

Q3 Do you think there might be a factor like compliance fatigue? (That is, people are more willing to comply with the measures and change their behaviour at the beginning and after some time, their willingness to comply may wear off)

Q4 Can you think of any barriers or facilitator to effective implementation of risk mitigation measures?

Q5 Looking forward, what are the takeaways? What can we do to better prepare the industry for emergencies like this?
